# A Control Strategy for Ground Fault on the AC Side of MMC-HVDC System

**DOI:** 10.1155/2022/1776209

**Published:** 2022-09-27

**Authors:** Hua Li, Liwei Guo

**Affiliations:** ^1^School of Energy and Power Engineering, Inner Mongolia University of Technology, Hohhot 010051, China; ^2^State Grid Zhangjiakou Electric Power Supply Company, Zhangjiakou 075000, China

## Abstract

This study explores a control strategy for a flexible Modular Multilevel Converter-High Voltage Direct Current (MMC-HVDC) system when there is an asymmetrical fault with the AC side system voltage. Aiming at the characteristic that the fault system has a negative sequence current path, this paper proposes a positive/negative sequence controller to ensure the output of original active power, and the validity of the proposed control strategy is verified by using simulation software PSCAD/EMTDC.

## 1. Introduction

The flexible DC system based on a modular multilevel converter (MMC) has the merits of modularized design and low harmonic wave content, and it is quite advantageous in terms of power supply reliability; it can access renewable energy conveniently and efficiently and realize multi-feed power supply and multi-infeed power reception. The technology has a very broad application prospect in future new energy grid connection, long-distance load power supply, microgrid power supply, urban DC distribution network, and other power transmission fields. Using MMC-HVDC to transmit new energy can alleviate the fluctuation and impact of intermittent and random renewable energy on the power grid, reduce the phenomenon of wind and light abandonment, and improve the utilization efficiency of renewable energy. It is an effective way to realize large-scale clean energy power transmission and grid connection [1].

Flexible DC technology is developing fast in China and the State Grid Corporation of China and China Southern Power Grid have successively built a series of demonstration projects. In 2011, the flexible DC project of Shanghai Nanhui Wind Farm was officially launched for commercial use, and its goal is to connect the Nanhui Wind Farm to the grid so that the low voltage ride-through (LVRT) capability of the wind farm is enhanced. This project is also the first flexible DC transmission project in Asia [2]. In 2013, a three-terminal flexible DC transmission project was initiated on Nan'ao Island in Shantou, Guangdong province. It is the first multiterminal flexible DC transmission project in the world, marking that China has become the first country in the world to fully master the whole series of core technologies for the design and test. This project adopted the MMC technology. It broke the monopoly of techniques and realized complete independent localization. For the internal interconnection systems of far offshore wind farms, this technology can save investment by more than 10% [3]. In 2014, a five-terminal flexible DC transmission project was officially launched in Zhoushan, Zhejiang province, this project has five converter stations and is equipped with an ocean transmission testing base, and it realized the mutual coordination of electric energy between the islands in Zhoushan and can provide a stable guarantee for the development of the Zhoushan area [4]. In 2015, a flexible DC transmission project was launched in Xiamen city, Fujian province. The project has two converter stations, Waipuyuan and Ludao; it strengthened the grid structure of the Xiamen area and can meet the local load growth demand [5]. In 2020, the Zhangbei flexible HVDC transmission project will have successfully become part of Beijing's power grid, and it is expected to deliver about 22.5 billion kilowatt-hours to Beijing each year. This project will not only serve the 2022 Winter Olympics but also greatly increase the proportion of new energy consumption in Beijing [6].

The research on flexible DC transmission projects started earlier in several foreign countries, and they have built electricity transmission projects with multiple MMC topological structures. For example, in 2011, Siemens' subsidiary in the United States finished the Trans Bay project, which realized a transmission length of 85 km and a transmission power of 400 MW [7]. In 2014, the HVDC underground transmission system that connected France and Spain was put into commercial operation, two 1000 MW.

Bipolar circuits were installed, and the transmission capacity between France and Spain was increased. The project improved the diversity of power sources and the safety of the power supply, and it enhanced the integration of the electricity market in southwestern Europe [8]. In order to achieve energy transformation, Germany has strengthened the use of wind power. Since the completion of the first offshore flexible DC line, BorWin1, Germany has signed a few North Sea flexible DC transmission wind power access projects, including BorWin2, HelWin1, and SylWin1. Currently, 40 percent of Germany's total renewable energy generation comes from wind power [9–11].

**Figure 1:**
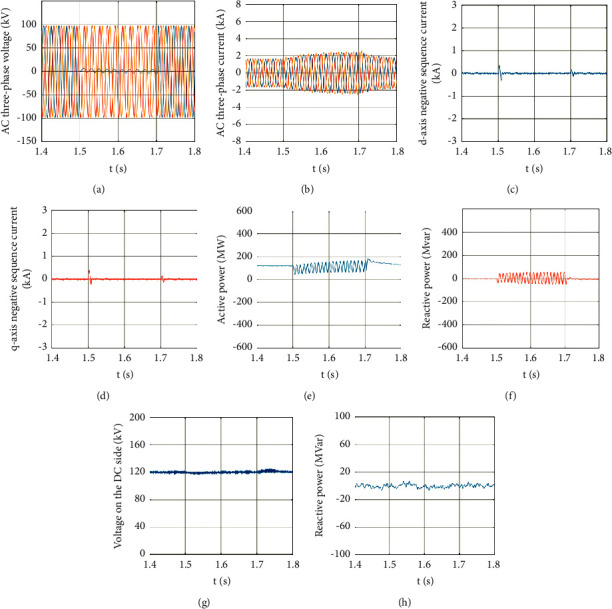
Control effect of single-phase ground fault. (a) Three-phase voltage on the fault side. (b) Three-phase current on the fault side. (c) *d*-axis negative sequence current on the fault side. (d) *q*-axis negative sequence current on the fault side. (e) Active power on the fault side. (f) Reactive power on the fault side. (g) DC voltage on the rectifier side. (h) Reactive power on the rectifier side.

According to the relative spatial location of the equipment, the faults of the MMC-HVDC system can be roughly divided into several types, including the AC-side faults of the converter station, the DC-side faults of converter station, and the internal faults of the converter station. Faults of various parts can cause failure to equipment in each region; in server cases, it can threaten the safety and stability of the entire MMC-HVDC system. In terms of current academic research on MMC control strategies, most studies aimed at the control strategy under a steady state, and few have been concerned with the control strategy under a transient state. For an MMC system, faults are mainly concentrated on the AC side, the DC side, and the inside of the converter station. No matter at which part the faults occur, the vehicle's ability to operate safely and stably will be greatly reduced. In nonstar delta connection transformer systems, zero-sequence current might show up. Li et al. [12] adopted a PR controller to suppress the zero-sequence and negative sequence currents in MMC-HVDC during asymmetric faults. In order to solve the bandwidth limitation of the traditional PR controller, Li et al. [13] used a quasi-PR controller to restrain the negative sequence current and active power ripple and improved the traditional voltage balance algorithm. Zhu et al. [14] proposed an improved voltage margin control strategy to quickly increase the grid voltage in the case of AC-side faults. Their method reduces the impact of DC voltage and can send out the corresponding reactive current. Wang et al. [15] proposed a fault control method based on the superposition of negative sequence voltages to solve the continuous operation ability of MMC under the condition of asymmetric faults, and their method improved the fault traversal ability of the system. In order to give an accurate quantitative analysis of the transient characteristics and circuit models of DC-side faults and short-circuit faults of the MMC-HVDC system, Yang et al. [16] explored the fault generation mechanism and gave the mathematical expressions of the fault voltage and current, according to the overvoltage and overcurrent phenomenon. According to Figures1(g) and 1(h), when the MMC2 side had the single-phase ground fault, the DC voltage and reactive power on the MMC1 side showed slight fluctuations, but on the whole, they remained stable. This paper attempts to construct a transient model for the asymmetrical condition of the AC system and obtain the type of harmonic waves of voltage and current after the fault occurs. This study also tries to design a control strategy for suppressing negative sequence current to improve the ability of the MMC-HVDC system to operate normally under asymmetrical fault conditions. Finally, the model was constructed on the PSCAD/EMTDC simulation platform, and the validity of the designed control strategy was demonstrated in this study.

## 2. Basic Topology of MMC

**Figure 2:**
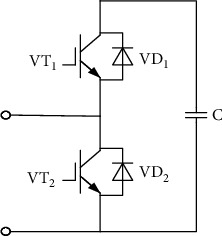
Basic structure of a half-bridge submodule.

**Figure 3:**
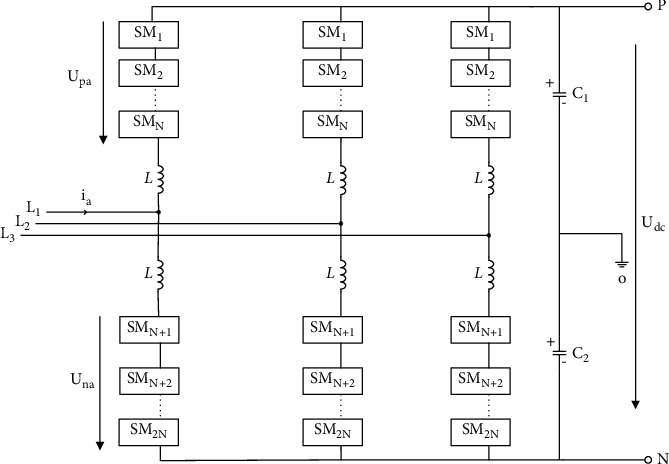
A basic modular multilevel topology.

This early MMC topology adopted the half-bridge submodule; this structure is simple and has good scalability. The basic structure of the early MMC is a half-bridge submodule structure.  Figure 2 shows the structure of the half-bridge submodule. However, when a short-circuit fault occurs on the DC side, it could not intercept the fault current. Moreover, capacitor charging and discharging can cause an increase in fault current, which can lead to safety problems for the equipment. If a relay protection device is used to clear the fault current, it will cause a short-term interruption to the power supply, causing problems such as damage to the freewheeling diode (FWD). In view of these problems, field scholars have researched many improved topological structures based on the topology of submodules and successively proposed the full-bridge submodule, single-clamp submodule, double-clamp submodule, improved composite submodule, and series double submodule, and all these topologies have a certain fault current blocking ability.  Figure 3 shows a basic modular multilevel topology; the MMC has 6 bridge arms, each of which contains 1 inductor and N cascaded submodules. It is characterized by realizing multilevel by connecting submodules in series, and its advantages are the number of levels is large, which greatly reduces the total harmonic distortion rate of the voltage; the switching frequency of a single device is relatively low; the stress of a single switching device is relatively low; a high degree of modularity is conducive to reducing costs; and so on. During normal operation, each bridge arm submodule is used to ensure the stability of the DC bus voltage to achieve the purpose of commutation. The number of submodules can be increased or decreased to satisfy application scenarios of different voltage levels and output power, and this cascading mode also helps to reduce the complexity and the investment cost of actual projects. On each bridge arm, besides the submodules, there is also an inductor *L*, and its purpose is to suppress the unbalanced current generated between bridge arms [17, 18].

MMC submodules have three common topologies: half-bridge, full-bridge, and cascaded two-level. The half-bridge submodule and the full-bridge submodule are totally identical in terms of main circuit, and their difference lies in the submodule composition. The number of IGBTs used in the full-bridge submodule is two times that of the half-bridge submodule. Correspondingly, the number of supporting equipment increases as well. The cascaded two-level submodule adopts the crimping-type processing method. It has both the functions of series voltage-sharing and capacitor voltage-sharing. The control and protection are quite complicated, and now in the world, only the products produced by ABB are relatively mature.

The performance comparison of the three converter topologies is shown in Table 1.

In terms of investment cost, the cost of the half-bridge submodule structure is lower and the working principle of the submodule is to control the on-off of IGBT. The submodule can present three working states of input, removal, and locking. During normal operation, the input and removal states of each submodule are controlled to realize the shaping of the AC side voltage. The structure is simple, the number of required devices is minimal, the overall loss is small, and the system efficiency is high. There are two output levels (0 and submodule capacitance voltage). In case of the event of a DC fault, it does not have the function of locking MMC.

Most of the current MMC-HVDC projects adopt this structure; therefore, this paper also selected the half-bridge structure, as shown in Figure 2. Each half-bridge submodule contains 2 IGBTs (VT_1_, VT_2_), 2 antiparallel diodes (VD_1_, VD_2_), and a capacitor. The capacitor voltage is used to maintain a constant DC side voltage [19].

## 3. Analysis of Fault Characteristics

### 3.1. A Mathematical Model of an AC System with an Asymmetric Fault

**Figure 4:**
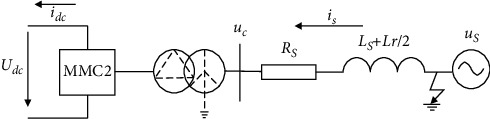
A schematic of the MMC-HVDC inverter side fault.

This paper mainly focuses on asymmetric faults. When an asymmetric fault occurs at a certain point in the power system, the symmetry condition of the three-phase circuit is destroyed, and the three-phase symmetrical circuit becomes asymmetrical. At this time, the symmetric component method can be used to turn the actual fault system into three mutually independent sequence component systems, and each sequence component system itself is three-phase symmetrical. Asymmetric fault types include single-phase ground faults, two-phase short-circuit, and two-phase short-circuit grounding, which are characterized by three-phase current asymmetry when an asymmetric fault occurs. Taking the MMC-HVDC inverter side as an example to analyze the status of the fault, the fault is simplified as shown in Figure 4.

In Figure 4, *u*_*s*_ is the AC system's three-phase voltage; *R*_*s*_ and *L*_*s*_ denote the equivalent resistance and inductance of the AC side; *L*_*r*_ represents the loss inductance of the bridge arm; the transformer adopted the star-delta connection mode; *i*_*dc*_ is the DC side current; and *U*_*dc*_ is the DC side voltage.

By analyzing the circuit in Figure 4, the KVL equation can be written as follows:

(1) $$\begin{array}{c} R_{s}i_{s}+\left(L_{s}+\frac{L_{r}}{2}\right)\frac{di_{s}}{dt}=u_{s}-u_{c}\text{.} \end{array}$$

By the symmetrical component method, it is known that *u*_*s*_, *u*_*c*_, and *i*_*s*_ can be decomposed into positive sequence components, negative sequence components, and zero-sequence components:

(2) $$\begin{array}{c} \left\{\begin{array}{c} u_{s}=u_{s1}+u_{s2}+u_{s0}\\ u_{c}=u_{c1}+u_{c2}+u_{c0}\\ i_{s}=i_{s1}+i_{s2}+i_{s0}\text{.} \end{array}\right. \end{array}$$

Because the transformer adopted the star-delta connection mode, the analysis did not take zero-sequence components into consideration; therefore, above formula can be simplified as follows:

(3) $$\begin{array}{c} \left\{\begin{array}{c} u_{s}=u_{s1}+u_{s2}\\ u_{c}=u_{c1}+u_{c2}\\ i_{s}=i_{s1}+i_{s2}\text{.} \end{array}\right. \end{array}$$

The three-phase stationary coordinate system is the ABC coordinate system and the stationary coordinates, and the three physical quantities in the three-phase stationary symmetrical coordinate system are 120° away from each other in the physical space angle. In a three-phase stationary coordinate system, Formula (1) can be decomposed into two subsystems of positive sequence and negative sequence, and the mathematical model is

(4) $$\begin{array}{c} \left\{\begin{array}{c} R_{s}i_{s1}+\left(L_{s}+\frac{L_{r}}{2}\right)\frac{di_{s1}}{dt}=u_{s1}-u_{c1}\\ R_{s}i_{s2}+\left(L_{s}+\frac{L_{r}}{2}\right)\frac{di_{s2}}{dt}=u_{s2}-u_{c2}\text{.} \end{array}\right. \end{array}$$

According to the equivalent principle of magnetomotive force in space, the transformation matrix from abc three-phase stationary coordinate system to dq rotating coordinate system is shown as follows:

(5) $$\begin{array}{c} T_{abc/dq}\left(\theta \right)=\frac{2}{3}\left[\begin{array}{ccc} \cos\;\theta & \cos\left(\frac{\theta -2\pi }{3}\right) & \cos\left(\frac{\theta +2\pi }{3}\right)\\ -\sin\;\theta & -\sin\left(\frac{\theta -2\pi }{3}\right) & -\sin\left(\frac{\theta +2\pi }{3}\right)\\ \frac{1}{2} & \frac{1}{2} & \frac{1}{2} \end{array}\right]\text{.} \end{array}$$

Let L *=* *Ls* *+* *Lr/*2, Formula (4) is transformed into the rotating coordinate system, and the transformed positive and negative sequence components are

(6) $$\begin{array}{c} \left\{\begin{array}{c} L\frac{di_{s\;d1}}{dt}=u_{s\;d1}+\omega_{0}Li_{sq1}-R_{s}i_{s\;d1}-u_{c\;d}\\ L\frac{di_{sq1}}{dt}=u_{sq1}-\omega_{0}Li_{s\;d1}-R_{s}i_{sq1}-u_{cq1}\text{,} \end{array}\right. \end{array}$$

(7) $$\begin{array}{c} \left\{\begin{array}{c} L\frac{di_{s\;d2}}{dt}=u_{s\;d2}-\omega_{0}Li_{sq2}-R_{s}i_{s\;d2}-u_{c\;d2}\\ L\frac{di_{sq2}}{dt}=u_{sq2}+\omega_{0}Li_{s\;d2}-R_{s}i_{sq2}-u_{cq2}\text{.} \end{array}\right. \end{array}$$

Formulas (6) and (7) are subject to Laplace transformation, and the mathematic model in the frequency domain is obtained as

(8) $$\begin{array}{c} \left\{\begin{array}{c} {\left(R_{s}+sL\right)i}_{s\;d1}\left(s\right)=u_{s\;d1}\left(s\right)+\omega_{0}Li_{sq1}\left(s\right)-u_{c\;d1}\left(s\right)\\ \left(R_{s}+sL\right)i_{sq1}\left(s\right)=u_{sq1}\left(s\right)-\omega_{0}Li_{s\;d1}\left(s\right)-u_{cq1}\left(s\right)\text{,} \end{array}\right. \end{array}$$

(9) $$\begin{array}{c} \left\{\begin{array}{c} \left(R_{s}+sL\right)i_{s\;d2}\left(s\right)=u_{s\;d2}\left(s\right)-\omega_{0}Li_{sq2}\left(s\right)-u_{c\;d2}\left(s\right)\\ \left(R_{s}+sL\right)i_{sq2}\left(s\right)=u_{sq2}\left(s\right)+\omega_{0}Li_{s\;d2}\left(s\right)-u_{cq2}\left(s\right)\text{.} \end{array}\right. \end{array}$$

**Figure 5:**
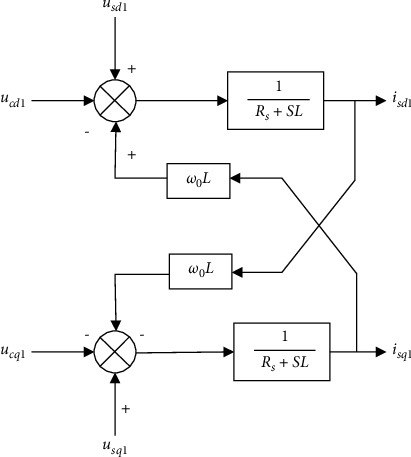
Positive sequence current controller.

**Figure 6:**
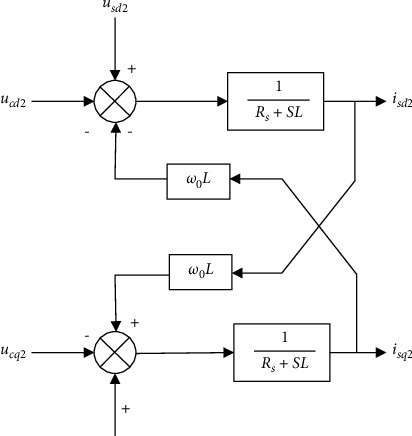
Negative sequence current controller.

The two formulas above show that the negative sequence components and the positive sequence components are symmetrical and independent of each other at this time, and their structures in the dq coordinate system can be drawn as Figures 5 and 6.

### 3.2. MMC Power Model of an AC System with an Asymmetric Fault

Based on the theory of instantaneous power, when the AC system has an asymmetric fault, the active power and reactive power received by MMC are

(10) $$\begin{array}{c} S=P+jQ=U_{S}I_{S}^{*}=\left(U_{S}^{+}e^{j\omega t}+U_{S}^{-}e^{j\omega t}\right)\left(I_{S}^{+}e^{j\omega t}+I_{S}^{-}e^{j\omega t}\right)\text{.} \end{array}$$

Substituting the positive and negative sequence components into Formula (10), we can get

(11) $$\begin{array}{c} \left\{\begin{array}{c} P=P_{0}+P_{S2}\sin\;\left(2wt\right)+P_{C2}\cos\;\left(2wt\right)\\ Q=Q_{0}+Q_{S2}\sin\;\left(2wt\right)+Q_{C2}\cos\;\left(2wt\right)\text{,} \end{array}\right. \end{array}$$

(12) $$\begin{array}{c} \left\{\begin{array}{c} P_{0}=\frac{3}{2}\left(u_{s\;d}^{+}i_{s\;d}^{+}+u_{sq}^{+}i_{sq}^{+}+u_{s\;d}^{-}i_{s\;d}^{-}+u_{sq}^{-}i_{sq}^{-}\right)\\ Q_{0}=\frac{3}{2}\left(u_{s\;d}^{+}i_{s\;d}^{+}-u_{sq}^{+}i_{sq}^{+}+u_{s\;d}^{-}i_{s\;d}^{-}-u_{sq}^{-}i_{sq}^{-}\right)\text{,} \end{array}\right. \end{array}$$

(13) $$\begin{array}{c} \left\{\begin{array}{c} P_{S2}=\frac{3}{2}\left(u_{s\;d}^{+}i_{s\;d}^{-}+u_{sq}^{+}i_{sq}^{-}+u_{s\;d}^{-}i_{s\;d}^{+}+u_{sq}^{-}i_{sq}^{+}\right)\\ Q_{S2}=\frac{3}{2}\left(u_{s\;d}^{+}i_{s\;d}^{-}-u_{sq}^{+}i_{sq}^{-}+u_{s\;d}^{-}i_{s\;d}^{+}-u_{sq}^{-}i_{sq}^{+}\right)\text{.} \end{array}\right. \end{array}$$

According to Formula (11), when the AC side has an asymmetric fault, active power and reactive power would have DC side components and double-frequency components, wherein the DC side components include *u*_*s* *d*_^+^*i*_*s* *d*_^+^, *u*_*sq*_^+^*i*_*sq*_^+^, *u*_*s* *d*_^−^*i*_*s* *d*_^−^, and *u*_*sq*_^−^*i*_*sq*_^−^ and the double-frequency components include *u*_*s* *d*_^+^*i*_*s* *d*_^−^, *u*_*sq*_^+^*i*_*sq*_^−^, *u*_*s* *d*_^−^*i*_*s* *d*_^+^, and *u*_*sq*_^−^*i*_*sq*_^+^. The double-frequency components can cause fluctuations in the DC side voltage, affecting its stability; when the fluctuating power flow is reversely transmitted to the opposite converter station, it will also cause fluctuations to the voltage and power in the DC side. Therefore, suppressing the double-frequency fluctuations is quite necessary (after the occurrence of an asymmetric fault, negative sequence components will be generated, which will affect the *P* and *Q* double-frequency fluctuations).

### 3.3. MMC-HVDC Control Strategy for Suppressing Negative Sequence Current of AC System

#### 3.3.1. Design of the Phase Sequence Decomposition Link

When the AC side of the MMC-HVDC system has an asymmetric fault, the phase detection link of the phase-locked loop (PLL) in the system needs to be changed. Now, field scholars have a few methods for extracting positive and negative sequences, including the instantaneous symmetrical component method, low-pass filtering (LPF) method, and numerical analysis method. For the purpose of making sure that the system can accurately lock phase and separate negative and positive sequence voltages and currents, this paper extracted the positive sequence voltage components from the unbalanced voltage first [20–22].

According to the instantaneous component method, the asymmetric sine components in the MMC-HVDC system were decomposed into positive sequence components and negative sequence components, which were then subjected to a 3/2 transformation to obtain the mathematical expression of the two-phase stationary coordinate system:

(14) $$\begin{array}{c} \left\{\begin{array}{c} \begin{array}{c} u_{S\alpha }=u_{S_{\alpha }}^{+}+u_{\alpha }^{-}=U^{+}\;\cos\;\left(\omega t+\varphi^{+}\right)\\ +U^{-}\;\cos\;\left(\omega t+\varphi^{-}\right) \end{array}\\ \begin{array}{c} u_{S\beta }=u_{S\beta }^{+}+u_{S\beta }^{-}=U^{+}\;\cos\;\left(\omega t+\varphi^{+}\right)\\ -U^{-}\;\cos\;\left(\omega t+\varphi^{-}\right), \end{array} \end{array}\right. \end{array}$$

where U^+^ and U^−^ stand for the amplitudes of the positive and negative sequence voltages and *φ*^+^ and *φ*^−^ stand for the initial phases of the negative and positive sequence voltages.

Shift the phase of Formula (14) by 90° to get

(15) $$\begin{array}{c} \left\{\begin{array}{c} \begin{array}{c} u_{s\alpha }^{s}=u_{s\beta }^{+}-u_{s\beta }^{-}=U^{+}\;\sin\;\left(\omega t+\varphi^{+}\right)\\ +U^{-}\;\sin\;\left(\omega t+\varphi^{-}\right) \end{array}\\ \begin{array}{c} u_{s\beta }^{s}=-u_{S\alpha }^{+}+u_{S\alpha }^{-}=U^{+}\;\cos\;\left(\omega t+\varphi^{+}\right)\\ +U^{-}\;\cos\;\left(\omega t+\varphi^{-}\right). \end{array} \end{array}\right. \end{array}$$

The relationship between positive sequence components and negative sequence components in the *αβ* coordinate system can be derived from Formulas (14) and (15):

(16) $$\begin{array}{c} \left[\begin{array}{c} u_{S\alpha }^{+}\\ u_{S\beta }^{+}\\ u_{S\alpha }^{-}\\ u_{S\beta }^{-} \end{array}\right]=M\left[\begin{array}{c} u_{S\alpha }\\ u_{S\beta }\\ u_{S\alpha }^{s}\\ u_{S\beta }^{s} \end{array}\right]\text{,} \end{array}$$

where *M* is as follows:

(17) $$\begin{array}{c} M=\left[\begin{array}{cccc} 1 & 0 & 0 & -1\\ 0 & 1 & 1 & 0\\ 1 & 0 & 0 & 1\\ 0 & 1 & -1 & 0 \end{array}\right]\text{.} \end{array}$$

Through the total components in the two-phase stationary coordinate system and the components after the 90° phase shift, according to formula (14)–(17) to measure the characteristics of component separation, the traditional positive and negative sequence separation method is adopted in this paper. The asymmetric positive and negative sequence components at the time of fault occurrence could be deduced, thereby achieving the accurate separation of positive sequence components.

According to the principle of coordinate transformation,

(18) $$\begin{array}{c} \theta =\omega t+\varphi^{+}\text{.} \end{array}$$

The expression in the dq synchronous rotating coordinate system can be obtained as

(19) $$\begin{array}{c} \left\{\begin{array}{c} \begin{array}{c} u_{s\;d}=U^{+}\;\cos\;\left(\omega t+\varphi^{+}-\theta \right)\\ +U^{-}\;\cos\;\left(\omega t+\varphi^{-}+\theta \right) \end{array}\\ \begin{array}{c} u_{sq}=U^{+}\;\sin\;\left(\omega t+\varphi^{+}-\theta \right)\\ -U^{-}\;\sin\;\left(\omega t+\varphi^{-}+\theta \right). \end{array} \end{array}\right. \end{array}$$

**Figure 7:**
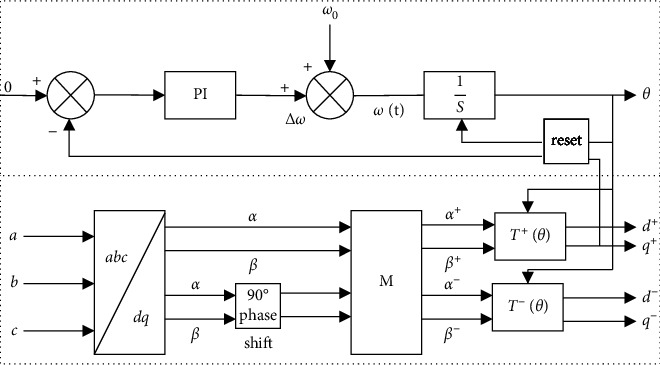
Detection of synchronous phases and symmetrical components.

Based on above analysis, the phase sequence decomposition link under unbalanced three-phase voltage condition could be drawn as the control block diagram shown in Figure 7.

$T^{+}\left(\theta \right)=\left[\begin{array}{cc} \cos\;\theta & \sin\;\theta \\ -\sin\;\theta & \cos\;\theta \end{array}\right]$ and $T^{-}\left(\theta \right)=\left[\begin{array}{cc} \cos\;\theta & -\sin\;\theta \\ \sin\;\theta & \cos\;\theta \end{array}\right]$.

### 3.4. Controller Design in Case of MMC-HVDC System Asymmetrical Fault

If the AC side of the MMC-HVDC system has an asymmetric fault, it will cause the three-phase current to be asymmetrical, and this is because the transformer has adopted a star/delta connection mode, so the fault current mainly includes negative sequence current and positive sequence current. When the asymmetric fault is not serious, it will trigger corresponding protection actions in the system; if the asymmetric fault is serious, it will threaten the operation safety of the converter stations at both ends and burn down related components.

For the purpose of reducing and avoiding the influence of asymmetrical current and the overvoltage and overcurrent caused by the fault and improving the ability of the entire system to operate uninterrupted in case of a fault event, this section designs a control strategy for suppressing negative sequence current by decoupling negative and positive sequence currents based on the transient mathematical model of the AC system, aiming to apply it to protect the equipment and guarantee that the system has the ability to operate stably and safely. Formulas (8) and (9) can be rewritten as

(20) $$\begin{array}{c} \left\{\begin{array}{c} \frac{di_{s\;d}^{+}}{dt}=-\frac{R}{L}i_{s\;d}^{+}+\omega i_{sq}^{+}-\frac{1}{L}u_{c\;d}^{+}+\frac{1}{L}u_{s\;d}^{+}\\ \frac{di_{sq}^{+}}{dt}=-\frac{R}{L}i_{sq}^{+}-\omega {\dot{i}}_{s\;d}^{+}-\frac{1}{L}u_{cq}^{+}+\frac{1}{L}u_{sq}^{+}\text{,} \end{array}\right. \end{array}$$

(21) $$\begin{array}{c} \left\{\begin{array}{c} \frac{di_{s\;d}^{-}}{dt}=-\frac{R}{L}i_{s\;d}^{-}-\omega i_{sq}^{-}-\frac{1}{L}u_{c\;d}^{-}+\frac{1}{L}u_{s\;d}^{-}\\ \frac{di_{sq}^{-}}{dt}=-\frac{R}{L}i_{sq}^{-}+\omega i_{s\;d}^{-}-\frac{1}{L}u_{cq}^{-}+\frac{1}{L}u_{sq}^{-}\text{.} \end{array}\right. \end{array}$$

Formulas (20) and (21) suggest that the *d*-axis remains to be coupled to the q-axis, by introducing the PI controller to the decoupling calculation; let

(22) $$\begin{array}{c} \left\{\begin{array}{c} \frac{di_{s\;d}^{+}}{dt}=\lambda \left(i_{s\;dr\;ef}^{+}-i_{s\;d}^{+}\right)\\ \frac{di_{sq}^{+}}{dt}=\lambda \left(i_{sqref}^{+}-\lambda i_{sq}^{+}\right)\text{,} \end{array}\right. \end{array}$$

(23) $$\begin{array}{c} \left\{\begin{array}{c} \frac{di_{s\;d}^{-}}{dt}=\mu \left(i_{s\;dr\;ef}^{-}-i_{s\;d}^{-}\right)\\ \frac{di_{sq}^{+}}{dt}=\mu \left(i_{sqref}^{-}-i_{sq}^{-}\right)\text{,} \end{array}\right. \end{array}$$

where *λ* and *μ* are parameters of the PI controller; *i*_*s* *dr* *ef*_^+^, *i*_*s* *dr* *ef*_^−^ and *i*_*sqref*_^+^, *i*_*sqref*_^−^ represent the positive sequence active current reference values of the *d*-axis and *q*-axis; by substituting above two formulas into Formulas (20) and (21), we can get

(24) $$\begin{array}{c} \left\{\begin{array}{c} u_{c\;d}^{+}=u_{s\;d}^{+}-Ri_{s\;d}^{+}+\omega Li_{sq}^{+}-\lambda L\left(i_{s\;dr\;ef}^{+}-i_{s\;d}^{+}\right)\\ u_{cq}^{+}=u_{sq}^{+}-Ri_{sq}^{+}-\omega Li_{s\;d}^{+}-\lambda L\left(i_{sqref}^{+}-i_{sq}^{+}\right) \end{array}\right.\\ \left\{\begin{array}{c} u_{c\;d}^{-}=u_{s\;d}^{-}-Ri_{s\;d}^{-}-\omega Li_{sq}^{-}-\mu L\left(i_{s\;dr\;ef}^{-}-i_{s\;d}^{-}\right)\\ u_{cq}^{-}=u_{sq}^{-}-Ri_{sq}^{-}+\omega Li_{s\;d}^{-}-\mu L\left(i_{sqref}^{-}-i_{sq}^{-}\right). \end{array}\right. \end{array}$$

**Figure 8:**
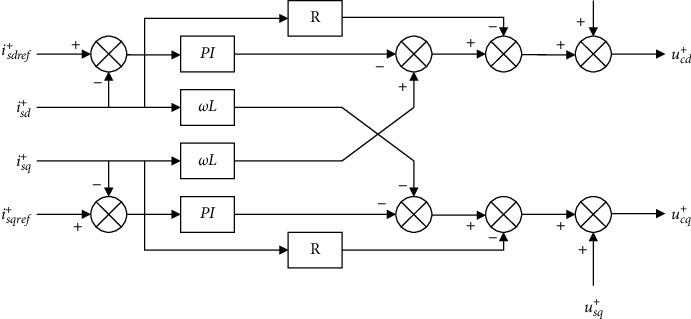
Structure of the inner loop decoupling controller for positive sequence systems.

**Figure 9:**
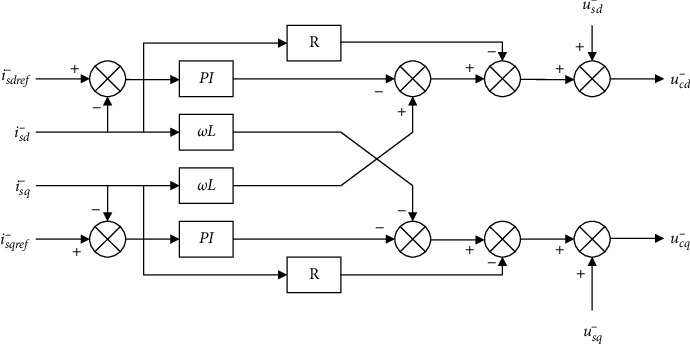
Structure of the inner loop decoupling controller for negative sequence systems.

According to the two formulas above, the decoupling control of positive and negative sequence currents in the current inner loop in the case of an asymmetric fault happening on the AC side of the MMC-HVDC system could be drawn as the schematics (Figures 8 and 9).

For the purpose of suppressing negative sequence current successfully, the reference values of the *dq* axis current of the negative sequence current in Formula (22) were set as

(25) $$\begin{array}{c} \left\{\begin{array}{c} i_{s\;de\;rf}^{-}=0\\ i_{sqref}^{-}=0\text{.} \end{array}\right. \end{array}$$

To improve the transmission ability of active power, it is significant to integrate the positive and negative sequence systems with the double closed-loop control on the inverter side to design the controller. The above formula was substituted into Formula (13) to obtain the relationship between the reactive power and active power reference values and the positive sequence current reference values:

(26) $$\begin{array}{c} \left[\begin{array}{c} P_{ref}\\ P_{ref} \end{array}\right]=\frac{3}{2}\left[\begin{array}{cc} u_{s\;d}^{+} & u_{sq}^{+}\\ u_{sq}^{+} & -u_{s\;d}^{+} \end{array}\right]\left[\begin{array}{c} i_{s\;de\;rf}^{+}\\ i_{sqerf}^{+} \end{array}\right]\text{.} \end{array}$$

**Figure 10:**
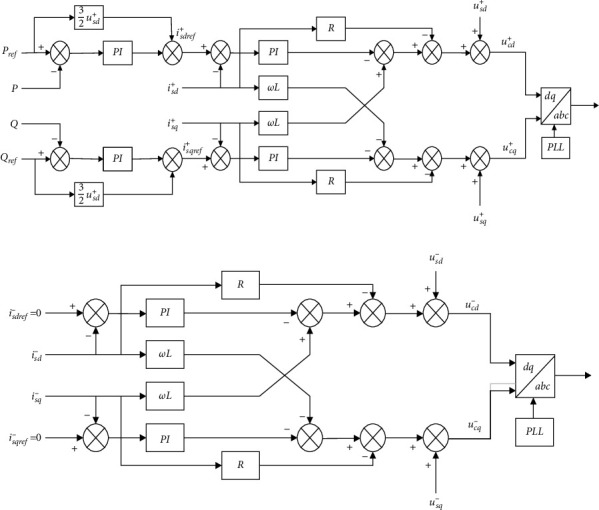
Control structure of the negative sequence current suppression on the inverter side in case of an asymmetrical fault with AC systems.

The above formula is the control strategy of the constant active power and constant reactive power of the outer loop of the negative sequence current suppressor. The reference values of the positive and negative sequence components of the active and reactive currents were taken as the reference values of the inner loop current controller. Combined with the negative and positive sequence decoupling link in Section 3.3.1, when the AC system has an asymmetric fault, the dual-loop control of negative sequence current suppression on the inverter side could be drawn as the schematic shown in Figure 10.

## 4. Simulation

### 4.1. Simulation Analysis of Single-Phase Ground Fault

**Figure 11:**
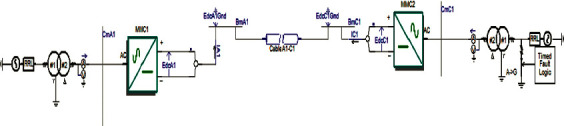
Simulation of an asymmetrical fault in the MMC-HVDC two-terminal system.

For the purpose of proving the effectiveness of the negative sequence current controller built in this study, an MMC-HVDC two-terminal system was constructed on the PSCAD/EMTDC simulation platform, as shown in Figure 11.

When the system had run for 1.5 s, a phase-A ground fault occurred on the AC-side bus of MMC2, the ground resistance was 0.001 Ω, fault duration was 0.2 s, and the fault was eliminated at 1.7 s. During the fault period, the reference values of the reactive power of the converters on both sides were both 0, and the transmission power of the non-fault phase remained unchanged during the fault period.  Figure 1 shows the control effect of the MMC-HVDC system fault and the recovery characteristics after the fault has been eliminated.

As can be seen from Figures 1(a) and 1(b), when an A-phase ground fault occurrs on the AC side of the MMC-HVDC system, the three-phase voltage is no longer symmetrical; because of the control strategy of suppressing negative sequence current, the AC side three-phase current keeps balance, but the three-phase current can remain symmetrical during the fault period. After the fault was eliminated, both the three-phase voltage and the three-phase current could restore to a balanced state. According to Figures 1(c) and 1(d), after the negative sequence current controller was added, the negative sequence current fluctuated greatly at the start and at the end of the fault, and it fluctuated around 0 during the fault period, indicating that the suppression effect of negative sequence current was obvious, and the negative sequence current controller designed in this paper had exerted an obvious effect when a fault occurred.

As can be seen from Figures 1(e) and 1(f), owing to the presence of the negative sequence voltage, the active power and reactive power on the fault side had certain double-frequency fluctuations. Since the fault point voltage does not fall to zero, the active power can still be transmitted. However, the active power can still be transmitted normally. Overall, the actual measured value remained near the reference value. According to Figures 1(g) and 1(h), when the MMC2 side had the single-phase ground fault, the DC voltage and reactive power on the MMC1 side showed slight fluctuations, but on the whole, they remained stable. The simulation shows that the negative sequence current is well suppressed and does not affect the power transmission between the two converter stations.

## 5. Conclusion

This paper first deduced the mathematical model of the MMC-HVDC system in the case of an asymmetric fault happening on the AC side. Then, based on the proposed model, this study designed a control strategy for suppressing negative sequence current through a phase sequence decomposition link, and the proposed strategy was proved to be able to enhance the ability of the MMC-HVDC system to operate normally under asymmetric fault conditions.

## Figures and Tables

**Table 1:** Performance comparison of three converter topologies.

Performance	Half-bridge MMC	Full-bridge MMC	Cascaded two-level converter
Scalability	Good	Good	Good
Switching frequency	Low	Low	Low
Harmonic wave content	Low	Low	Low
DC fault suppression	Have not	Have	Have not
Reactive power adjustment range	High	Very high	High

## Data Availability

The data used to support the findings of this study are available from the corresponding author upon request.
